# Effects of Low-Intensity and Long-Term Aerobic Exercise on the Psoas Muscle of *mdx* Mice: An Experimental Model of Duchenne Muscular Dystrophy

**DOI:** 10.3390/ijms23094483

**Published:** 2022-04-19

**Authors:** Emilly Sigoli, Rosangela Aline Antão, Maria Paula Guerreiro, Tatiana Oliveira Passos de Araújo, Patty Karina dos Santos, Daiane Leite da Roza, Dilson E. Rassier, Anabelle Silva Cornachione

**Affiliations:** 1 Department of Physiological Sciences, Federal University of São Carlos (UFSCar), Rod. Washington Luís, km 235-SP-310-São Carlos, São Paulo 13565-905, Brazil; emillysigoli@estudante.ufscar.br (E.S.); rosangelaaline@estudante.ufscar.br (R.A.A.); mpguerreiro@estudante.ufscar.br (M.P.G.); tatiana.passos@ufscar.br (T.O.P.d.A.); pattysantos@estudante.ufscar.br (P.K.d.S.); 2 Department of Neurosciences and Behaviour, Ribeirão Preto Medical School, University of São Paulo-Ribeirão Preto, São Paulo 14051-160, Brazil; daianeroza@hotmail.com; 3 Department of Kinesiology and Physical Education, McGill University, Montreal, QC H2W 1S4, Canada; dilson.rassier@mcgill.ca

**Keywords:** Duchenne muscular dystrophy, *mdx* mice, satellite cells, PGC-1α, low-intensity aerobic exercise, immunofluorescence

## Abstract

Duchenne muscular dystrophy (DMD) is a muscle disease characterized by the absence of the protein dystrophin, which causes a loss of sarcolemma integrity, determining recurrent muscle injuries, decrease in muscle function, and progressive degeneration. Currently, there is a need for therapeutic treatments to improve the quality of life of DMD patients. Here, we investigated the effects of a low-intensity aerobic training (37 sessions) on satellite cells, peroxisome proliferator-activated receptor-gamma coactivator (PGC)-1α protein (PGC-1α), and different types of fibers of the psoas muscle from *mdx* mice (DMD experimental model). Wildtype and *mdx* mice were randomly divided into sedentary and trained groups (*n* = 24). Trained animals were subjected to 37 sessions of low-intensity running on a motorized treadmill. Subsequently, the psoas muscle was excised and analyzed by immunofluorescence for dystrophin, satellite cells, myosin heavy chain (MHC), and PGC-1α content. The minimal Feret’s diameters of the fibers were measured, and light microscopy was applied to observe general morphological features of the muscles. The training (37 sessions) improved morphological features in muscles from *mdx* mice and caused an increase in the number of quiescent/activated satellite cells. It also increased the content of PGC-1α in the *mdx* group. We concluded that low-intensity aerobic exercise (37 sessions) was able to reverse deleterious changes determined by DMD.

## 1. Introduction

Duchenne muscular dystrophy (DMD) is the most common and severe muscle disorder caused by a mutation in chromosome X (region Xp 21.2) that leads to an absence of the protein dystrophin [1]. DMD affects one in every 5000 boys, and clinical manifestations appear in childhood, causing muscle weakness and the loss of the ability to walk, wheelchair confinement, and respiratory and cardiac failure [1,2]. Dystrophin is part of a protein complex and provides stability to muscle fibers during muscle contractions. With the absence of dystrophin, lesions in the sarcolemma occur, resulting in weakness and loss of muscle function [3,4].

The psoas is a glycolytic muscle, predominantly composed of type II fibers (FTII), and one of the most susceptible to injuries in DMD [5,6]. The preferential involvement of glycolytic fibers in DMD may be directly related to different cellular and biochemical characteristics, such as the presence/quantity of satellite cells (SCs) and peroxisome proliferator-activated receptor-gamma coactivator (PGC)-1α protein (PGC-1α), as both are found in greater amounts in oxidative muscles when compared with glycolytic muscles [7,8].

The SCs are responsible for the regeneration of muscle fibers. Even inactive, muscles express Pax7, and, in the presence of muscle injury, the SCs are activated and migrate to the injury site, where they proliferate due to the high expression of MyoD and Myf-5. Then, differentiation occurs, marked by downregulation of Pax7 and upregulation of Mrf4 and myogenin [9,10]. In DMD, SCs may promote a deregulation of the regeneration–degeneration cycle. PGC-1α is responsible for increasing the number of mitochondria and increasing the oxidative metabolism, and it has an important role in angiogenesis, inflammation, and formation of neuromuscular junctions. The expression of PGC-1α can be induced by several physiological factors, such as exercise and fasting [11,12]. In DMD, the capacity for oxidative phosphorylation and mitochondrial functions are reduced, likely modulated by the expression of the PGC-1α protein [8].

Currently, there is no cure for DMD, but it is essential to evaluate potential treatments aimed at providing a better quality of life and delaying disease progression for patients with DMD [13]. Low-intensity physical exercise has been studied in rehabilitation programs, but the benefits of exercise for DMD are still controversial [14,15,16]. Studies performed with *mdx* mice, an experimental model for DMD, have shown that low-intensity physical training can improve muscle function and strength, morphology, and resistance to fatigue, which opens promising possibilities for improving the quality of life of patients with DMD [17,18,19].

Physical exercise in DMD can increase the number of SCs in rodents submitted to both voluntary wheel training and forced treadmill running [20]. Training also increases the expression of PGC-1α [21] and the expression of utrophin [22]. In this study, we investigated if low-intensity training on a treadmill, applied for a long period of time (37 sessions), could improve the morphology and cause positive adaptations in the psoas muscle of *mdx* mice. The *mdx* mouse model is a well-established experimental model of DMD [23]. We observed that 37 sessions of training reverted some of the deleterious aspects caused by muscle dystrophy, improving several morphologic characteristics of the psoas muscle through SC activation and an increase in PGC-1α protein content. Our study provides new information regarding the benefits of long and low-intensity aerobic exercise on the dystrophic muscle, representing a noninvasive therapeutic strategy for the treatment of DMD.

## 2. Results

### 2.1. Morphological Characteristics of the Dystrophic Muscle Improved after Low-Intensity Exercise

**Figure 1:**
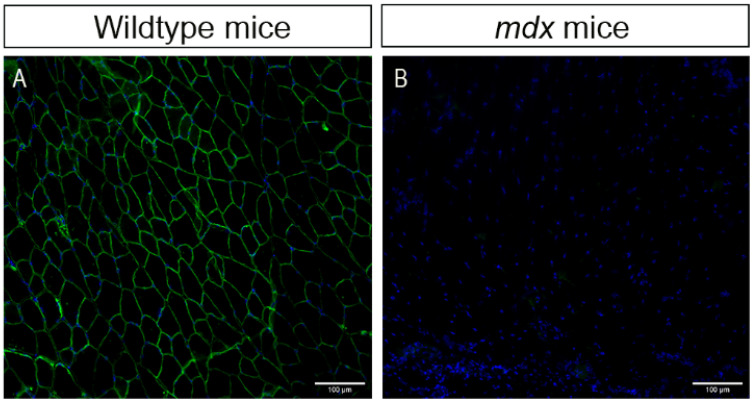
Dystrophin protein immunostaining in the psoas muscle of mice. (**A**) Wildtype mice (control); dystrophin present in green. (**B**) *mdx* mice (dystrophic); dystrophin absent. Cell nuclei were stained with DAPI (blue). Images were obtained with the ImageXpress XLS System microscope at 20× magnification. Scale bar = 100 µm.

We used an immunofluorescence assay to confirm the absence of the dystrophin protein in *mdx* animals (Figure 1). We observed the presence of dystrophin in the sarcolemma of psoas fibers in wildtype mice (green color, Figure 1A) and the absence of the protein in the muscle of *mdx* mice (Figure 1B), as expected.

**Figure 2:**
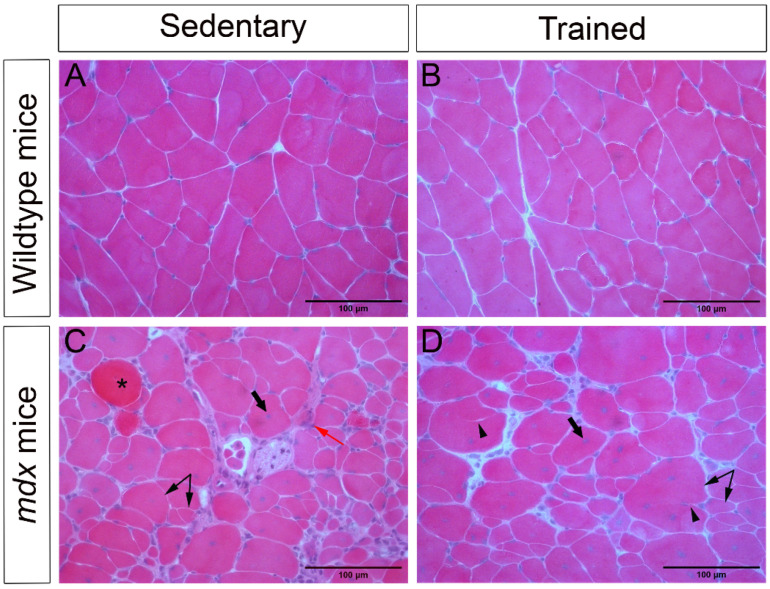
Low-intensity exercise improved the morphological characteristics of the dystrophic muscle. (**A**) Normal cytoarchitecture of the psoas muscle of WT-SED mice. (**B**) Normal cytoarchitecture of the psoas muscle of WT-TR mice. (**C**) Pathological changes in the psoas muscle of *mdx*-SED mice. (**D**) Exercise improved the tissue cytoarchitecture of *mdx*-TR mice, although pathological characteristics were still observed. Asterisk: basophilic cell; thick arrow: centralized nucleus; red arrow: necrosis/inflammatory infiltrate; arrowhead: splitting; double arrow: variation in fiber size. Scale bar = 100 µm.

A semiquantitative morphological analysis of the psoas muscle stained with hematoxylin and eosin (HE) showed a healthy muscle structure, with polyhedral fibers, peripheral nuclei, and no pathological changes in the WT-SED and WT-TR groups (Figure 2A,B). However, we observed variation in fiber size, centralized nuclei (an indicative of muscle degeneration and regeneration), basophilic fibers, splitting, necrosis, increased connective tissue, and inflammatory infiltrate in the *mdx*-SED group (Figure 2C). The *mdx*-TR group presented similar alterations, but they were less expressive, which resulted in an improvement in the muscle cytoarchitecture (Figure 2D, Table 1).

### 2.2. Low-Intensity Exercise Promotes an Increase in the Number of Satellite Cells in the Dystrophic Muscle

**Figure 3:**
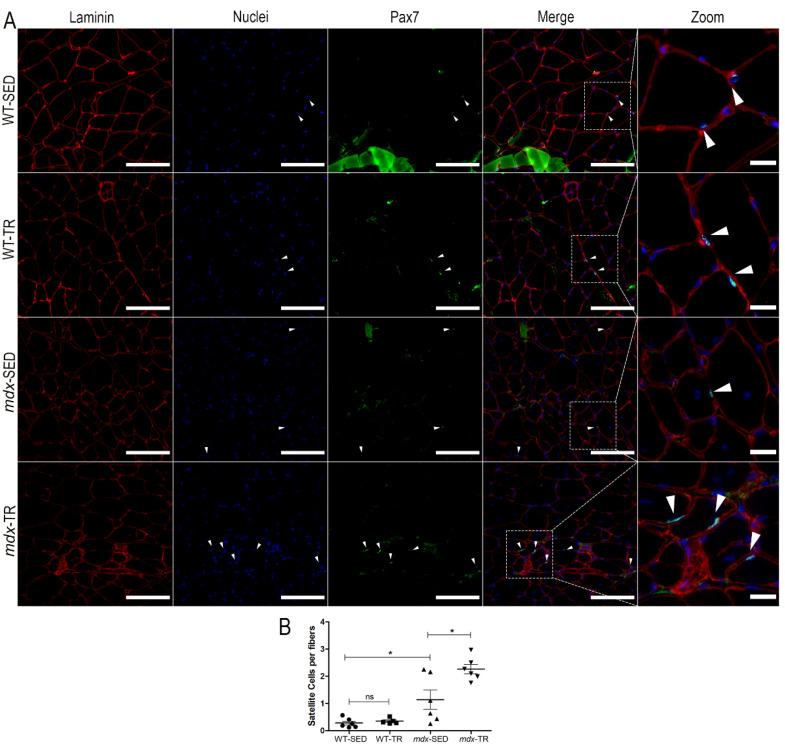
Satellite cell number (Pax7 marker) is increased in dystrophic muscles after 37 sessions of low-intensity exercise. (**A**) Representative images of immunostaining of quiescent/activated satellite cells through the Pax7 antibody. Laminin (red, Cy5), nuclei (blue, DAPI), and Pax7 (green, FITC) are shown. White arrows: Indication of SCs (Pax7 + underlying nuclei). Images were obtained with the ImageXpress XLS System microscope at 40× magnification. Scale bar = 100 µm (laminin, nuclei, Pax7 and merged panels). All zoomed areas provided better visualization of the SCs (scale bar = 20 µm). SCs were identified by their localization below the laminin superimposed with the nuclei. (**B**) As expected, the WT-SED group had few quiescent/activated SCs when compared to the *mdx*-SED group. After a low-intensity training for 37 sessions, the *mdx*-TR group showed increased SC content when compared to the *mdx*-SED group. * *p* < 0.05; ns: not significant. Round symbol: WT-SED; Square symbol: WT-TR; Triangle symbol: *mdx-*SED; Inverted triangle symbol: *mdx*-TR. Abbreviations: WT-SED: sedentary wildtype; WT-TR: trained wildtype; *mdx*-SED: sedentary *mdx*; *mdx*-TR: trained *mdx*.

Quiescent/activated SCs were identified using the Pax-7 marker (Figure 3A). The WT-SED group had few quiescent/activated SCs when compared to the *mdx*-SED group (WT-SED × *mdx*-SED, *p* < 0.05) (Figure 3B). After training, we observed an increase in the SC number in *mdx* mice when compared to the respective sedentary group (*mdx*-SED × *mdx*-TR, *p* < 0.05) (Figure 3B).

**Figure 4:**
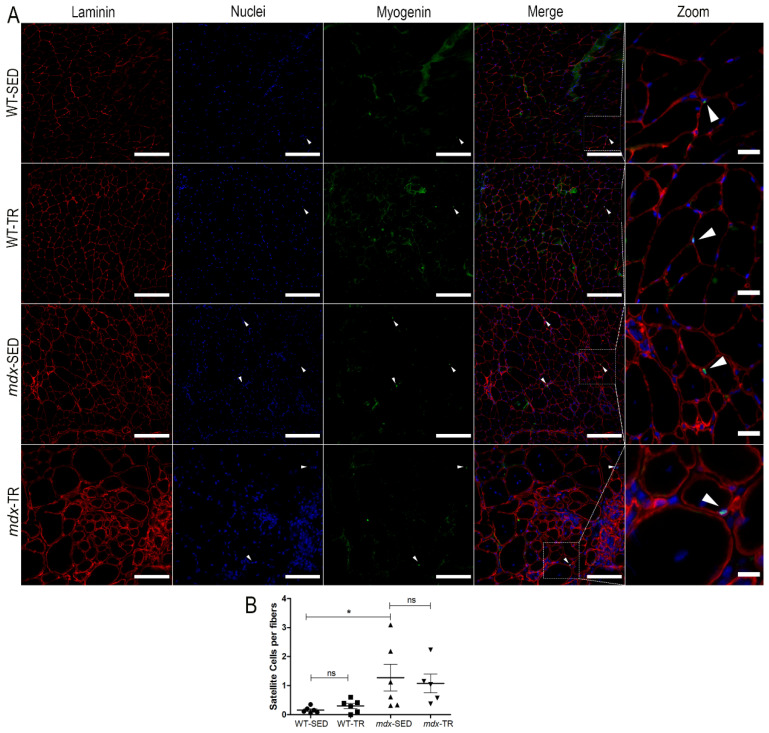
Myogenin (satellite cells) content is increased in sedentary *mdx* mice. (**A**) Representative images of immunostaining of SC in differentiation/fusion through the F5D antibody (myogenin). Laminin (red, Cy5), nuclei (blue, DAPI), and myogenin (green, FITC) are shown. White arrows: Indication of SCs (F5D + underlying nuclei). Images were obtained with the ImageXpress XLS System microscope at 20× magnification. Scale bar = 100 µm (laminin, nuclei, myogenin, and merged panels). All zoomed areas provided better visualization of the SCs (scale bar = 30 µm). SCs were identified by their localization below the laminin superimposed with the nuclei. (**B**) As expected, the WT-SED group had fewer SCs in differentiation/fusion when compared to the *mdx*-SED group. * *p* < 0.05, ns: not significant. Round symbol: WT-SED; Square symbol: WT-TR; Triangle symbol: *mdx-*SED; Inverted triangle symbol: *mdx*-TR. Abbreviations: WT-SED: sedentary wildtype; WT-TR: trained wildtype; *mdx*-SED: sedentary *mdx*; *mdx*-TR: trained *mdx*.

Additionally, SCs in differentiation/fusion were identified using F5D, a myogenin marker (Figure 4A). As expected, the WT-SED group had fewer SCs in differentiation/fusion when compared to the *mdx*-SED group (WT-SED × *mdx*-SED, *p* < 0.05) (Figure 4B).

### 2.3. PGC-1α Is Increased in Dystrophic Muscles

**Figure 5:**
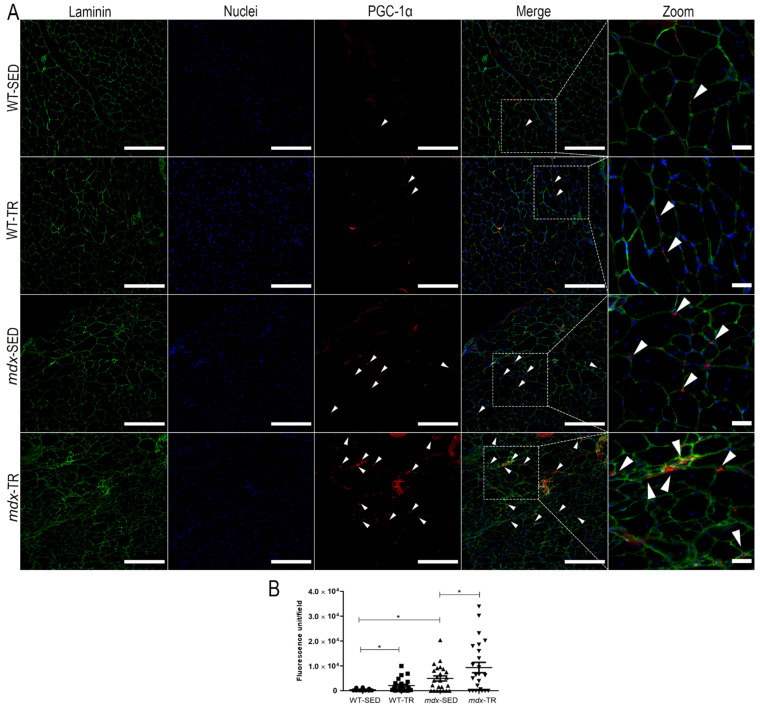
PGC-1α content is increased in dystrophic muscles. (**A**) Representative images of immunostaining of PGC-1α. Laminin (red, Cy5), nuclei (blue, DAPI), and PGC-1α (green, FITC) are shown. White arrows: Indication of PGC-1α. Images were obtained with the ImageXpress XLS System microscope at 20× magnification. Scale bar = 100 µm. All zoomed areas provided better visualization of the PGC-1α (scale bar = 30 µm). (**B**) *mdx*-SED group showed higher expression of PGC-1α when compared to the WT-SED group. After low-intensity training (37 sessions), the WT-TR and *mdx*-TR groups showed increased expression of PGC-1α when compared to the sedentary animals. * *p* < 0.05. Round symbol: WT-SED; Square symbol: WT-TR; Triangle symbol: *mdx-*SED; Inverted triangle symbol: *mdx*-TR. Abbreviations: WT-SED: sedentary wildtype; WT-TR: trained wildtype; *mdx*-SED: sedentary *mdx*; *mdx*-TR: trained *mdx*. PGC-1α: Peroxisome proliferator-activated receptor-gamma coactivator (PGC)-1α protein.

Fluorescence measurements for the PGC-1α protein showed that DMD increased the oxidative characteristics of the psoas muscle, as we observed a higher amount of the protein in the *mdx*-SED group when compared to the WT-SED group (WT-SED × *mdx*-SED, *p* < 0.05) (Figure 5A,B). As expected, the low-intensity aerobic training, applied for a long period, increased the amount of PGC-1α in both trained groups (WT-TR and *mdx*-TR) when compared to their respective sedentary groups (Figure 5A,B).

### 2.4. A Long Period of Low-Intensity Aerobic Exercise Increases the Trophism of Fibers in mdx Mice

Fiber diameter analyses showed a significant reduction in the trophism of FTIIA, FTIID, and FTIIB fibers of the psoas muscle of the *mdx*-SED group when compared to the animals in the WT-SED group (WT-SED × *mdx*-SED, *p* < 0.05) (Table 2). However, after 37 training sessions, it was possible to observe an increase in FTIIB and FTIID diameters in the *mdx*-TR group when compared to the sedentary one (*mdx*-SED × *mdx*-TR, *p* < 0.05) (Table 2). On the other hand, in WT animals, training reduced the diameter of FTIIB and FTIIDB/BD when compared to the WT-SED group (WT-SED × WT-TR, *p* < 0.05) (Table 2).

### 2.5. Low-Intensity Exercise Did Not Affect the Fiber Type Distribution

**Figure 6:**
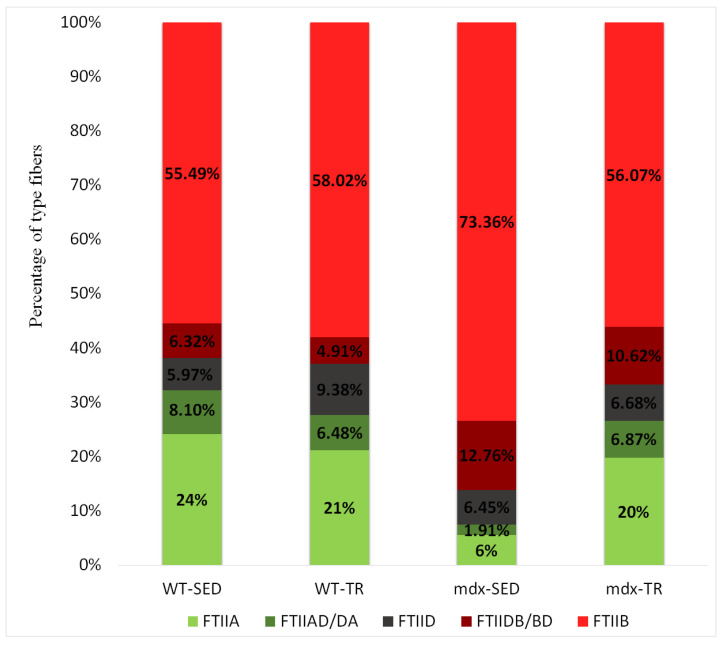
Percentage of type IIA, IIAD/DA, IID, IIDB/BD, and IIB muscle fibers in all groups studied. There was no change in the number of different fiber types. Abbreviations: WT-SED: sedentary wildtype; WT-TR: trained wildtype; *mdx*-SED: sedentary *mdx*; *mdx*-TR: trained *mdx*.

**Figure 7:**
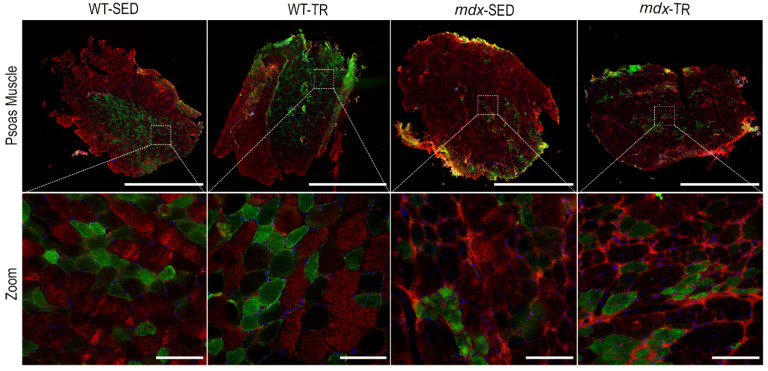
Representative images of immunostaining of different fiber types. MHC IIB (red, Cy5), nuclei (blue, DAPI), and MHC IIA (green, FITC) are shown. Images were obtained with the ImageXpress XLS System microscope at 4× magnification. Scale bar = 5000 µm (psoas muscle panels). All Zoomed areas at 40× magnification provided better visualization of the types of fibers in the psoas muscle (scale bar = 100 µm). Abbreviations: WT-SED: sedentary wildtype; WT-TR: trained wildtype; *mdx*-SED: sedentary *mdx*; *mdx*-TR: trained *mdx*.

As expected, we observed a predominance of FTIIB fibers in all studied groups, as the psoas is a glycolytic muscle characterized by the dominance of this fiber type (Figure 6 and Figure 7). The presence of hybrid fibers (FTIIAD/DA and FTIIDB/BD) was also verified (Figure 6 and Figure 7). However, 37 training sessions were not able to change the number of different fiber types.

## 3. Discussion

In the present study, we confirmed that the absence of dystrophin in *mdx* mice causes negative alterations in the psoas muscle. Most importantly, after 37 sessions of low-intensity aerobic training on a treadmill, we observed an improvement in several characteristics of the dystrophic muscle, an indicative that physical exercise, when applied correctly, can be an important and promising noninvasive therapeutic tool to treat DMD. We observed a decrease of basophilic cells, a decrease in inflammatory markers, and few necrotic areas in the muscle of trained *mdx* mice, which indicates that the exercise improved the general characteristics of the muscle cytoarchitecture. Our data corroborate several studies that also observed an improvement in the morphology of *mdx* mice muscles after a low-intensity exercise training [24,25].

A better understanding of the regenerative capacity of the *mdx* mice muscle can provide important information about the disease; thus, it is important to investigate the role of satellite cells in muscle regeneration, from the quiescence and activation phases to the differentiation and fusion phases, where the process of repair and formation of new myofibers takes place [9,10]. We analyzed SCs through Pax7 (quiescence–activation) and myogenin (differentiation–fusion) fluorescent markers. As expected, we observed a large number of SCs in the psoas of the sedentary *mdx* animals (Pax-7^+^; myogenin^+^), a consequence of the chronic muscle damage that leads to numerous degeneration-regeneration cycles, activating SCs for repair [26,27]. This high number of SCs suggests that these cells are still able to participate in muscle repair, although they are dysfunctional due to the absence of dystrophin [28]. Ribeiro Jr and colleagues (2019) demonstrated that dystrophic gastrocnemius muscles retain a pool of proliferating SCs that rapidly respond to regenerating stimuli and that the muscle repair is abnormal, but preserved by upregulation of myogenic factors such as myogenin, a result that corroborates our observations [29].

It is known that aerobic exercise activates SCs and improves their function in healthy muscles, both in humans and in rodents [21,30,31]. Our results showed that dystrophic mice, after low-intensity training for 37 sessions, had an increase in SC activation (Pax7^+^) when compared to the sedentary animals. Interestingly, we did not observe an increase in differentiated SCs (Myog) after the applied exercise protocol, which suggests that some fibers were already repaired after training (low expression of myogenin by SCs). Myogenin is a muscle regulatory factor, temporarily expressed during differentiation; thus, data interpretation is temporally limited [32,33]. Yablonka-Reuveni and Anderson (2006) also reported an acceleration in the differentiation of SC in *mdx* mice when compared to normal muscles, a result that was supported by the significant improvement in the cytoarchitecture of the muscle tissue [34], which corroborates our findings.

We also demonstrated that the disease increased the oxidative characteristics of dystrophic muscles, as we observed high amounts of PGC-1α in the sedentary *mdx* mice when compared to the wildtype animals. This was an unexpected result, considering that PGC-1α regulates mitochondria biogenesis, which helps to compensate for the metabolic dysregulation present in dystrophic muscles [35]. Moreover, skeletal muscles of DMD individuals have a reduced capacity for oxidative phosphorylation, in addition to mitochondrial dysfunction [22,36]. It is still unclear why we found an increased PGC-1α content in the psoas muscle of the sedentary *mdx* mice, and future studies are needed to elucidate this finding.

The low-intensity aerobic training (37 sessions) increased PGC-1α protein levels in both trained groups (wildtype and *mdx* trained animals) when compared to their respective sedentary groups. Studies have shown that physical exercise induces the expression of PGC-1α, mainly in chronically exercised muscles, and that overexpression of the protein increases mitochondrial biogenesis, improving the oxidative capacity in muscles [8,37,38]. In the case of *mdx* mice, the overexpression of PGC-1α can improve muscle function, reducing the negative effects of the pathology [21,39,40,41]. It is known that PGC-1α induces utrophin expression, a protein homologous to dystrophin in dystrophic muscles, which compensates for its absence, being a promising strategy for the treatment of DMD [42,43]. Therefore, the increased PGC-1α content found in the psoas muscles of trained *mdx* animals in this study is an important and promising result that indicates the role of PGC-1α as a protective factor on dystrophic muscles.

DMD also led to a significant reduction in the diameter of pure fibers (FTIIA, FTIID, and FTIIB) in the psoas muscles of the sedentary *mdx* group (*mdx*-SED), suggesting the occurrence of an atrophic process during the disease progression, as reported in the literature [44,45]. After low-intensity training (37 sessions), we observed an increase in the diameter of FTIID and FTIIB fibers in the psoas muscles of the *mdx* animals (*mdx*-TR). This result has clinical relevance, helping to prevent the progression of muscle injuries, since glycolytic fibers (FTIIB, FTIID) can support mechanical loads imposed during muscle contraction [46]. It is well known that PGC-1α protects myofibers, and its overexpression increases the mitochondrial content and resistance to fatigue, reducing muscle atrophy in cases of denervation [47,48]. Although we did not perform an analysis to elucidate the association among fiber types, trophism, and PGC-1α protein expression, our data suggest that the increase in PGC-1α content had a positive effect on the trophism of glycolytic fibers. Consequently, we hypothesize that the augmented diameter of glycolytic fibers characterizes muscle protection in the psoas of the trained *mdx* mice. In addition, aerobic exercise also plays a role in preventing skeletal muscle atrophy and may serve as a prior treatment for atrophic situations, such as in DMD disorder, which presents intense atrophy over time [49].

The psoas of mice contains type IIA, IID, and IIB fibers unevenly distributed throughout the muscle, with a region containing only FTIIA, FTIID, and FTIIB fibers and a region composed almost exclusively of FTIIB [50,51,52]. Our results showed this predominance of FTIIB in all groups, as well as an unequal distribution of fibers. We also identified the presence of hybrid fibers (IIAD/DA and IIDB/BD), suggesting a combination of myosin heavy chains (MHC) that can undergo transformation from one fiber type to another (shifting) [53]. Glycolytic fibers are the most affected by DMD, undergoing more degenerative processes than oxidative fibers [54,55]. Lindsay and colleagues (2019) proposed that the resistance of oxidative fibers occurs due to the increased expression of utrophin [56], and Selsby and colleagues (2012) observed that overexpression of PGC-1α induces a shift from glycolytic (fast-twitch) to oxidative (slow-twitch) fibers in dystrophic muscles [57]. We know that physical exercise can increase the oxidative capacity of all myofibers, promoting a transition from more glycolytic to more oxidative fibers [58]. This shift has already been observed in different muscles of *mdx* mice after physical training [24,59,60]. However, studies using the psoas muscle of *mdx* mice are scarce. Although our results did not show statistical differences in the proportion of fibers between the trained and sedentary *mdx* animals, the decrease in the number of FTIIB fibers concomitantly with an increase in FTIIA suggests a positive adaptation of the psoas muscle. It is tempting to speculate that glycolytic muscles need mechanical stimulation of low intensity for a longer period than 37 sessions of training to present a significant shift in the proportion of muscle fibers.

One limitation of the present study is that we did not characterize the number of SCs in different fiber types, as it is known that oxidative fibers have more satellite cells than glycolytic cells [7,54,61]. However, given the high proportion of type II fibers in the psoas, we believe that our results are representative of what happens with DMD before and after training of the whole muscle.

## 4. Materials and Methods

### 4.1. Animals

This study was approved by the Ethics Committee on Animal Use of the Federal University of São Carlos-UFSCar (protocol number-CEUA n° 4740230518). Twelve wildtype mice (C57BL-10) and 12 *mdx* mice (C57BL-10; experimental model of DMD) were purchased from the Multidisciplinary Center for Biological Investigation on Laboratory Animal Science (CEMIB, UNICAMP, Campinas, Brazil). All animals were maintained in cages supplied with water and food ad libitum in an environment with a light–dark cycle (12 h/12 h) at 22 °C. Twenty-four male mice were used in this study. Twelve *mdx* mice (C57BL/10-Dmd*mdx*/PASUnib; body weight 18.33 ± 1.49 g) were randomly divided into two experimental groups: *mdx*-SED (sedentary *mdx; n* = 6) and *mdx*-TR (trained *mdx; n* = 6), and 12 wild-type mice (background: C57BL/10; body weight 19 g ± 0.0 g) were randomly divided into two experimental groups: WT-SED (sedentary wildtype; *n* = 6) and WT-TR (trained wildtype; *n* = 6).

### 4.2. Low-Intensity Training

Training sessions started when the mice completed 6 weeks. Before each session, all animals underwent a 2 min warm-up period, at a speed of 7 m/min. During the training period, they exercised at a speed of 9–10 m/min for 30 min. Current studies show that these values correspond to low-intensity exercise for *mdx* mice [18,62]. In total, 37 training sessions were carried out on a motorized treadmill (EP 132C; Insight, Ribeirão Preto, São Paulo, Brazil). The training was performed 3 days per week (Monday, Wednesday and Friday), until completing 37 training sessions (12 weeks and 1 day) to investigate muscle adaptations after a long period of training. Pedrazzani and colleagues (2021) showed that low-intensity training proved to be more effective when applied over longer periods [24]. The animals were sacrificed after the end of the 37th session (WT-SED, WT-TR, *mdx*-SED and *mdx*-TR) at 18 weeks old and 1 day, and the psoas muscle was excised and frozen in liquid nitrogen for histological and immunofluorescence observation.

### 4.3. Histology

Histological sections of frozen psoas muscles (6 μm of thickness) were obtained with a cryostat (Leica CM 1850 UV, Wetzlar, Hessen, Germany) at −25 °C. Hematoxylin and eosin (HE) stain was used to evaluate the morphological alterations of the muscle fibers of wildtype and *mdx* mice (centralized nucleus, basophilic cells, necrosis, and presence of inflammatory infiltrate). Images were captured using a light microscope Zeiss Vert.A1, software AxioVision Rel 4.8 (40x lens) (Zeiss, Jena, Thuringia, Germany), and a semiquantitative analysis was performed.

### 4.4. Immunofluorescence

Immunofluorescence was applied to quantify satellite cells during activation and differentiation, as well as PGC-1α protein, different isoforms of myosin heavy chain (MHC), and dystrophin protein. Slides containing the frozen psoas muscles were blocked with M.O.M. (mouse on mouse, Vector Laboratories, Burlingame, CA, USA). They were then incubated in primary antibodies with the following concentrations: anti-dystrophin (1:400; ab15277; Abcam, Cambridge, UK), anti-laminin (1:200; ab11575; Abcam, Cambridge, UK), anti-Pax-7 (1:10; sc-81648; Santa Cruz Biotechnology, Dallas, TX, USA), anti-PGC-1α (1:50; sc-518025; Santa Cruz Biotechnology, Dallas, TX, USA), F5D-myogenin (1:1; ab-2146602; DSHB-Developmental Studies Hybridoma Bank, Iowa City, IA, USA), MHC type 2A (SC-71, 1:50; ab-2147165; DSHB, Iowa City, IA, USA), and MHC type 2B (BF-F3, 1:100; ab-2266724; DSHB, Iowa City, IA, USA) in 1% BSA (Bovine serum albumin, Sigma Aldrich, San Luis, MO, USA) for 45 min at 37 °C. The slides were washed with PBS and incubated in secondary antibodies with the following concentrations: Alexa Fluor^®^ 488-green (1:200; ab-143165; Invitrogen, Waltham, MA, USA), Alexa Fluor^®^ 488-green (1:200; 115-545-205, Jackson Immuno Research, West Grove, PA, USA), Alexa Fluor^®^ 647-red (1:200; ab-2535812, Invitrogen, Waltham, MA, USA), Alexa Fluor^®^ 647-red (1:1000; sc-24637; Santa Cruz Biotechnology, Dallas, TX, USA), and Alexa Fluor^®^ 647-red (1:500; ab-150123, Abcam, Cambridge, UK). Slides were mounted in FluoroQuest™ Mounting Medium with 4′,6-diamidino-2-phenylindole (DAPI, nuclei staining; cat#20004; AAT Bioquest^®^; Sunnyvale, CA, USA). Immunofluorescent images were analyzed with the ImageXPress XLS System microscope (Molecular Devices, San Jose, CA, USA) (magnifications 4×, 10×, 20×, and 40×). The minimal Feret’s diameters and proportion of fiber types were analyzed in four random fields (size 1630.35 × 1630.35 μm; magnification 4×) using Image J software (version 1.50e, NIH, Bethesda, MD, USA) [24]. The minimal Feret’s diameters were measured with the minimum distance of the parallel tangents in opposing borders of all muscle fibers in each field. The proportions of fibers were analyzed in the same four fields where all fiber types were counted. The PGC-1α content was quantified from the fluorescence unit, and all satellite cells, located below the laminin staining underlying the nucleus, were counted. Using the same images, all muscle fibers were counted for calculation of the SC/fiber ratio. All analysis was done using the Image J software (version 1.52a, Bethesda, MA, USA) [63].

### 4.5. Statistical Analysis

Quantitative comparisons for immunofluorescence of Pax-7, myogenin, and PGC-1α were made through a one-way analysis of variance (ANOVA), followed by post hoc analysis using Bonferroni or Brown–Forsythe and Welch tests. The analyses were performed with GraphPad Prism (version 5.01 for Windows, San Diego, CA, USA). Data for the minimal Feret’s diameter and the proportion of different fiber types were analyzed using mixed-effects linear models, and multiple comparisons with contrasts were performed using the SAS software (version 9.4; SAS Institute Inc., Cary, NC, USA). The proportion of fibers was analyzed after a natural logarithmic transformation of the data to obtain homogeneous variability, consistent with a normal distribution [64]. Values of *p* < 0.05 were considered statistically significant for all analyses.

## 5. Conclusions

Low-intensity aerobic exercise applied for 37 sessions reversed some of the deleterious alterations determined by DMD, improving tissue morphology and fiber trophism through SC activation and increased content of PGC-1α protein in the psoas muscle of *mdx* mice. Our results suggest that a chronically applied low-intensity aerobic exercise can be a noninvasive therapeutic modality to improve or delay the degeneration of dystrophic muscles in individuals with DMD without side-effects.

## Figures and Tables

**Table 1:** Semiquantitative analysis of pathological changes in the psoas fibers identified by hematoxylin and eosin.

Pathological Changes/Groups	WT-SED (%)	WT-TR (%)	*mdx*-SED (%)	*mdx*-TR (%)
Nuclear centralization	50 *	83 *	100	100
Splitting	16 *	66 *	100	100
Variation in size	-	66	100	100
Basophilic fibers	-	-	83 *	66 *
Necrosis	-	-	100 *	100 *
Increased connective tissue	-	16 *	33 *	50 *

Abbreviations: WT-SED: sedentary wildtype; WT-TR: trained wildtype; *mdx*-SED: sedentary *mdx*; *mdx*-TR: trained *mdx*. The percentage refers to the number of mice that presented the anomaly in the group. * <5% of the cells.

**Table 2:** Mean values of minimal Feret’s diameters (μm) and the respective 95% confidence intervals (bottom rows) in psoas type IIA, type IIAD/DA, type IID, type IIDB/BD, and type IIB fibers in the different groups studied.

	WT-SED	WT-TR	*mdx*-SED	*mdx*-TR
FTIIA	24.95 *	24.36	20.00	18.96
	24.40–25.50	23.72–25.01	18.67–21.32	18.08–19.84
FTIIAD/DA	29.63	29.93	25.07	21.81
	28.46–30.80	28.44–31.42	21.30–28.84	20.36–23.25
FTIID	27.12 *	25.33	21.34	25.76 *
	25.87–28.37	24.38–26.28	19.82–22.85	24.21–27.30
FTIIDB/BD	29.96	25.94 †	27.09	29.63
	28.50–31.42	24.23–27.45	25.63–28.55	27.82–31.45
FTIIB	35.51 *	33.46 †	32.06	34.29 *
	34.92–36.11	32.90–34.02	31.36–32.75	33.42–35.15

* *p* < 0.05 compared to *mdx*-SED; † *p* < 0.05 compared to WT-SED. Abbreviations: WT-SED: sedentary wildtype; WT-TR: trained wildtype; *mdx*-SED: sedentary *mdx*; *mdx*-TR: trained *mdx*.

## Data Availability

All data generated or analyzed during this study are included in this published article.

## Abbreviations

DAPI	4′,6-Diamidino-2-phenylindole
DMD	Duchenne muscular dystrophy
FTI	Type I fibers
FTII	Type II fibers
HE	Hematoxylin and eosin
IF	Immunofluorescence
*mdx*	Experimental model of Duchenne muscular dystrophy
MHC	Myosin heavy chain
MOM	Mouse on mouse
PBS	Phosphate-buffered saline
PGC-1α	Peroxisome proliferator-activated receptor-gamma coactivator-1α
SC	Satellite cell
WT	Wildtype
